# High-Definition DIC Imaging Uncovers Transient Stages of Pathogen Infection Cycles on the Surface of Human Adult Stem Cell-Derived Intestinal Epithelium

**DOI:** 10.1128/mbio.00022-22

**Published:** 2022-02-01

**Authors:** Jorik M. van Rijn, Jens Eriksson, Jana Grüttner, Magnus Sundbom, Dominic-Luc Webb, Per M. Hellström, Staffan G. Svärd, Mikael E. Sellin

**Affiliations:** a Science for Life Laboratory, Department of Medical Biochemistry and Microbiology, Uppsala University, Uppsala, Sweden; b Department of Cell and Molecular Biology, Uppsala University, Uppsala, Sweden; c Department of Surgical Sciences, Uppsala University, Uppsala, Sweden; d Department of Medical Sciences, Gastroenterology and Hepatology Unit, Uppsala University, Uppsala, Sweden; UC Berkeley

**Keywords:** *Giardia*, *Salmonella*, human, infection dynamics, intestine, live-cell imaging, organoid, enteroid, polarized epithelia

## Abstract

Interactions between individual pathogenic microbes and host tissues involve fast and dynamic processes that ultimately impact the outcome of infection. Using live-cell microscopy, these dynamics can be visualized to study, e.g., microbe motility, binding and invasion of host cells, and intrahost-cell survival. Such methodology typically employs confocal imaging of fluorescent tags in tumor-derived cell line infections on glass. This allows high-definition imaging but poorly reflects the host tissue’s physiological architecture and may result in artifacts. We developed a method for live-cell imaging of microbial infection dynamics on human adult stem cell-derived intestinal epithelial cell (IEC) layers. These IEC layers are grown in apical imaging chambers, optimized for physiological cell arrangement and fast, but gentle, differential interference contrast (DIC) imaging. This allows subsecond visualization of both microbial and epithelial surface ultrastructure at high resolution without using fluorescent reporters. We employed this technology to probe the behavior of two model pathogens, Salmonella enterica serovar Typhimurium and Giardia intestinalis, at the intestinal epithelial surface. Our results reveal pathogen-specific swimming patterns on the epithelium and show that Salmonella lingers on the IEC surface for prolonged periods before host cell invasion, while Giardia uses circular swimming with intermittent attachments to scout for stable adhesion sites. The method even permits tracking of individual Giardia flagella, demonstrating that active flagellar beating and attachment to the IEC surface are not mutually exclusive. This work describes a generalizable and relatively inexpensive approach to resolving dynamic pathogen-IEC layer interactions, applicable even to genetically nontractable microorganisms.

## INTRODUCTION

Although infectious diseases of the intestine are often caused by large populations of invading pathogens, disease progression and outcome are ultimately dictated by the interactions of individual microbes with the host tissues. To characterize the complex dynamics of these underlying interactions, live-cell microscopy has become the method of choice. However, it is intrinsically difficult to study dynamic microbe interactions with internal host tissues such as the intestinal epithelium *in vivo*. The resolution of *ex vivo*-based microscopy techniques often suffers from the complexity and depth of intact tissues, resulting in the need for phototoxic high-dosage illumination or complex and expensive 2-photon setups. Therefore, researchers have turned to transformed or immortalized cell lines to study intestinal epithelial infections in cultured proxies of the gut epithelium. These cell lines often fail to recapitulate key features of intestinal epithelial cell (IEC) layers, such as a densely packed polarized morphology, a microvilliated apical surface, and sensitivity to cell death mechanisms, but have nevertheless uncovered a wealth of information about pathogen infection cycles (1–6). In contrast, the impact of physiologically relevant host cell and tissue parameters on infection dynamics remains understudied.

In the past decade, cultured organoid models have been shown to provide a powerful intermediate for this physiological gap between cell lines and intact primary tissues. The central features of the gut epithelium are faithfully recapitulated in both intestinal organoids derived from pluripotent stem cells (PSCs) (7, 8) and in so-called enteroids or colonoids derived from adult epithelial stem cells (ASCs) of small intestine or colon, respectively (9, 10). Organoid models can be cultured in a variety of two- and three-dimensional (2D and 3D) settings (11, 12) and retain nontransformed cell behavior over time (13, 14).

Despite their potential, organoid models have so far only been sparsely used for live-cell imaging of intestinal infection processes (15–17). In our experience, this is a result of the difficulty of adapting current imaging approaches from cell line infections to accommodate the properties of physiologically grown organoid-derived epithelia. First, while cell lines can be grown flat on glass cultureware to exploit the optimal working distance and numerical aperture of microscope objectives, intestinal organoid-derived IECs only develop into their natural polarized arrangement when cultured within rich extracellular matrices (ECMs) or atop permeable supports. The latter can be accommodated by ECM-coated transwell inserts with permeable membranes of polyethylene terephthalate (PET) or similar polymers as a 2D substrate (18, 19). Enteroid/colonoid-derived IEC layers in such transwell inserts can be efficiently cultured, differentiated, and infected by a variety of gut pathogens (20–28) but are poorly compatible with live-cell imaging. Second, nontransformed cells are difficult to manipulate genetically, which complicates introduction of fluorescent tags to visualize the host cell and its subcellular architecture by fluorescence microcopy. Finally, in contrast to tumor-derived cell lines, nontransformed cells retain sensitive cell death and stress signaling pathways, which makes them susceptible to phototoxicity and other perturbations and introduces a need for gentle imaging conditions. Taken together, these constraints have limited the applicability of live-cell imaging to characterize encounters of pathogens with the apical portion of nontransformed IEC layers at high spatial and temporal resolution.

Here, we present a method to visualize microbial infection cycle dynamics at the apical surface of ASC-derived IEC layers. This setup abolishes the need for (genetic) fluorescent markers, reduces phototoxicity, and approaches a physiologically arranged host cell-microbe interphase. To overcome the imaging constraints introduced by PET transwells, we developed a method to grow microvilliated human gut epithelium layers on ECM-coated alumina membranes in 3D-printed holders, which we denote apical imaging chambers (AIC). In addition, we overcame the need for fluorescent tagging and high-dose illumination by optimizing the conditions for high-resolution, live differential interference contrast (DIC) microscopy. The use of DIC rather than more phototoxic fluorescent reporter approaches favors physiological pathogen and host cell behavior and allows simultaneous visualization of both individual microbes and IEC surface ultrastructure without channel switching. We used this method to map the infection cycles of two prominent human gut pathogens atop the epithelial surface: the Gram-negative bacterium Salmonella enterica serovar Typhimurium and the protozoan Giardia intestinalis. Salmonella causes foodborne infections, resulting in diarrhea and fever, and relies on the fecal-oral route. Once ingested, Salmonella invades IECs in the host’s distal small intestine or proximal colon to induce inflammation and establish colonization (1, 29). Giardia also infects through the fecal-oral route and is found as a hardy cyst in the natural environment. Upon ingestion, these cysts transform into flagellated trophozoites that cause diarrhea, nausea, and intestinal cramping (30). Giardia trophozoites attach to the intestinal epithelium of the proximal small intestine but do not invade host cells. Both microbes, however, rely on flagellar motility for successful infection and spread. Using our newly developed AIC approach, we describe previously unrecognized single-microbe behaviors during IEC attachment for both pathogens. We show that this imaging methodology enables detailed, dynamic studies of pathogen and host cell behaviors at the interface of gut infection and should be adaptable to any culturable microbial species.

## RESULTS

### An apical imaging chamber enables high-definition live-cell DIC microscopy at the surface of intestinal epithelial cell layers.

**FIG 1:**
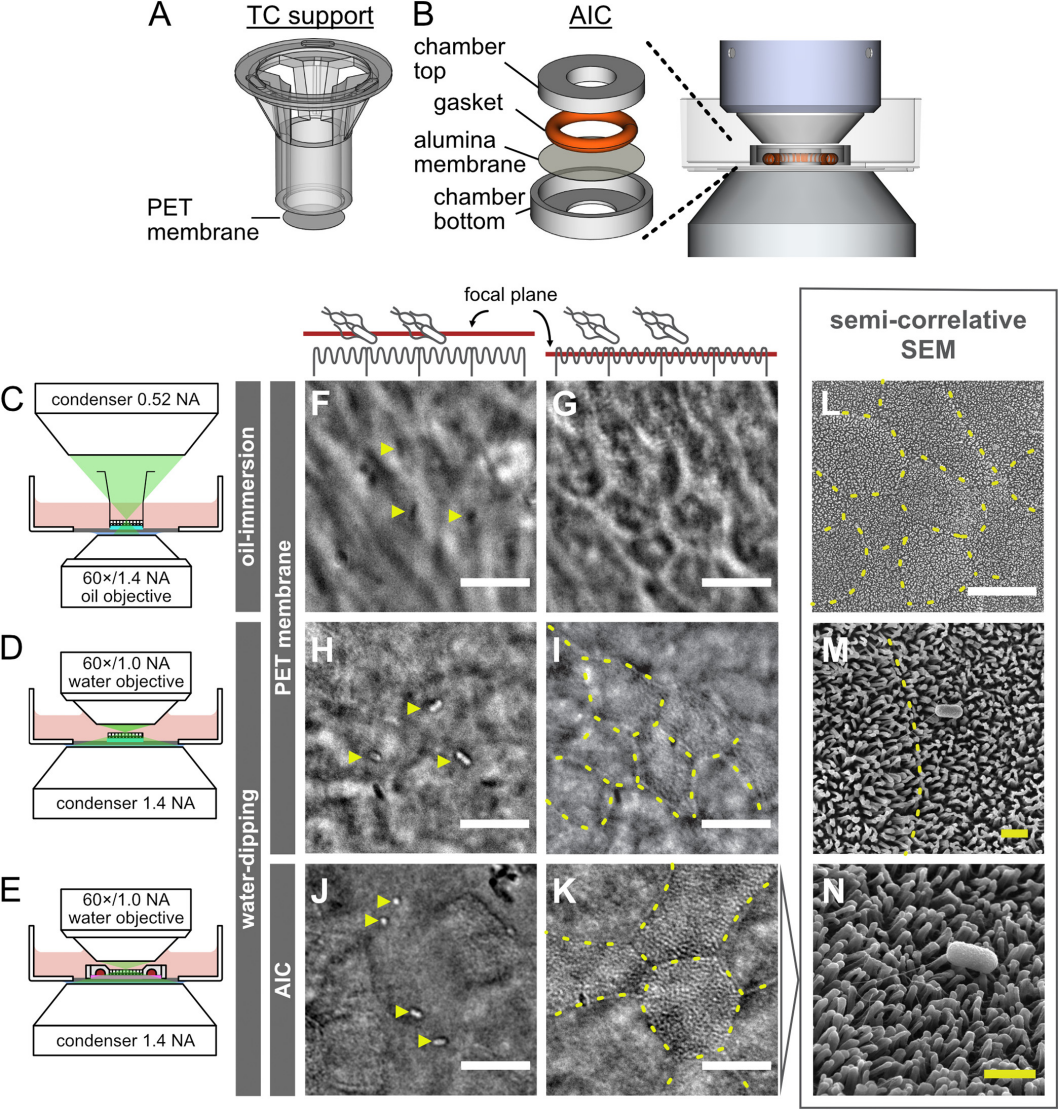
High-definition live-cell DIC microscopy of IEC monolayer infections in a novel apical imaging chamber. Schematic comparison of assemblies for IEC monolayer culture highlight that PET transwell membrane holders have a large plastic support (A), which has been omitted from the novel AICs (B). Both structures were placed in a 35-mm glass-bottom dish for imaging. The lower height of AICs allows for closer proximity and, thus, greater numerical aperture of the microscope’s objective and condenser. The optical interference caused by the large plastic transwell support and the resulting larger working distances of both objective and condenser is illustrated by live-cell imaging of infections in intact PET transwells in an inverted microscope (C, F, and G) versus cutout PET membranes using an upright water-dipping microscope (D, H, and I). This change in microscope setup improved the lateral resolution of bacteria (Salmonella) on the cell surface (F and H) and the apical surface topology (G and I) of IECs. Either bacteria or surface topology could be emphasized by slightly changing the focal plane, as indicated schematically on top. Replacing the PET membrane with an alumina membrane held within the custom-designed AIC (E) further improved the imaging resolution and minimized optical interference (J and K). AICs allow for sequential imaging of the same sample with DIC and SEM (L to N). The latter confirmed that differentiated IECs grown in AICs exhibit a highly interconnected epithelium with a densely microvilliated surface. Bacteria are indicated with yellow arrowheads. Overlaid dashed lines indicate cell-cell junctions. White and yellow scale bars are 10 and 1 μm, respectively.

We developed a method for live-cell imaging of microbe interactions with nontransformed human ASC-derived intestinal epithelial cell layers, aiming to combine (i) high structural definition, (ii) high temporal resolution, (iii) gentle imaging conditions to avoid phototoxicity and the need for fluorescent reporters, and (iv) a confluent, polarized IEC arrangement. To achieve this, we sought to improve upon the constraints presented by PET transwell supports to DIC imaging (Fig. 1A). Specifically, the PET membrane depolarizes light, the pores in the membranes diffract light, and the plastic membrane holder prevents close approach of the microscope objective to the apical side of the IECs, thereby constraining the working distance and numerical aperture. Therefore, a suitable alternative to PET membranes should not introduce optical interference for imaging within the visual spectrum but, like PET membranes, should be permeable to permit efficient epithelial cell polarization. In addition, the membrane holder should allow close proximity of both the objective and condenser to the epithelial surface to optimize the numerical aperture of the system.

This led us to evaluate membranes of anodized aluminum oxide (alumina) as a candidate substrate. Alumina forms a dense honeycomb-like structure with parallel, subdiffraction limit sized pores (see Fig. S1A in the supplemental material) and is optically transparent when wet. Unlike for PET membranes, the pores cannot be distinguished by light microscopy and the alumina does not depolarize transmitted light (Fig. S1B). Although alumina membranes poorly support cell adhesion, various adhesion-enhancing surface modifications have been reported for this material (31–33). We found that surface hydroxylation followed by sequential coating with poly-l-lysine and Matrigel enabled efficient attachment and expansion of human ASC-derived IECs atop the alumina membranes (Fig. S1C; see Materials and Methods for details). Furthermore, IECs grown on coated alumina membranes developed into confluent, highly polarized monolayers, reflecting *in vivo* epithelium architecture (Fig. S1D). To hold the alumina membrane in place during cell culture, we designed a 3D-printable plastic chamber (Fig. 1B). The design of the chamber can easily be adapted to alternative applications, and design files are freely available for download (https://doi.org/10.17044/scilifelab.16402539). Like a regular transwell, the chamber creates a customizable cell culture area in the middle, but the height of the chamber was kept slim to match the working distance of a water-dipping objective, thereby removing air-liquid interfaces within the light path. In addition, the chamber allows the objective and especially the condenser to be placed in close proximity to the sample, maximizing the utilization of the condenser's numerical aperture and, thus, improving the lateral resolution of DIC imaging.

10.1128/mbio.00022-22.2 FIG S1 Characterization of human IEC monolayers grown within AICs (supplement to Fig. 1). (A) The alumina membranes that were used feature densely packed pores with a diameter of ∼0.2 μm, as shown by SEM (scale bar, 1 μm). A major advantage of the alumina membranes versus the standard PET membranes is the lack of interference with polarized light. As shown in panel B, PET membranes affect the polarization of light, while alumina membranes do not interfere with the light’s polarity and are in this characteristic indistinguishable from glass. (C) Although alumina membranes do not intrinsically support IEC monolayer culture, surface treatment with H_2_O_2_ and poly-l-lysine (PLL) and a subsequent protein coat of Matrigel (MG) enable attachment of enteroid-derived IECs. (D) When enteroid-derived IECs are grown and differentiated on coated alumina membranes, a confluent, polarized, and microvilliated IEC monolayer is formed. The z-stack in panel D shows the microvilliated surface (red frame), cell-cell junctions (yellow frame), supernucleus height (green frame), equally distributed nuclei in a continuous plane (cyan frame), and basal stress fibers (blue frame). Nuclei are stained with DAPI and F-actin with phalloidin-AF488. Scale bars, 10 μm. Download FIG S1, TIF file, 2.9 MB. Copyright © 2022 van Rijn and Eriksson et al. 2022 van Rijn and Eriksson et al. https://creativecommons.org/licenses/by/4.0/ This content is distributed under the terms of the Creative Commons Attribution 4.0 International license.

To test if this apical imaging chamber (AIC) and an upright microscope setup indeed improved the quality of live DIC imaging of epithelial infection, we compared this system to the existing PET transwell supports. Human jejunal IEC monolayers were grown and differentiated atop PET transwells, or in AICs, and the apical compartment was infected with wild-type Salmonella. The PET transwells were imaged using either a standard inverted DIC microscope and an oil immersion objective (Fig. 1C) or the PET membrane cut out from the holder for imaging with the upright water-dipping objective setup (Fig. 1D). Infections in the AICs were imaged in parallel using the same upright system (Fig. 1E). As expected, the contrast and resolution of both the apical IEC surface and the Salmonella was poor for PET transwells imaged through the inverted microscope (Fig. 1F and G) but markedly improved through the water-dipping upright system (Fig. 1H and I). However, residual optical interference from the PET membrane was still evident, resulting in image blurring. Moreover, the need to cut out the PET membrane prior to imaging complicated sample handling, increased risk of mechanical cell damage, and caused the loose membrane to float with convection currents in the imaging medium, which prevented stable image acquisition over time.

The AIC markedly improved on all these imaging issues by providing a stable surface, minimizing the optical interference from membrane pores, and allowing easy handling underneath the water-dipping objective. As such, DIC imaging of live, infected IEC monolayers in AICs showed distinct contrast of Salmonella atop the cell surface (Fig. 1J). When focusing on the IEC surface itself, we observed clearly demarcated cell-cell junctions and a remarkable roughness made up of contrasting punctae (Fig. 1K), suggestive of a densely microvilliated surface. To correlate the live DIC image with the IEC surface topology, we disassembled noninfected and Salmonella-infected AICs and imaged the epithelial monolayers therein by scanning electron microscopy (SEM). This provided a powerful, semicorrelative setup, as SEM analysis could be done on the same AIC samples used for live DIC. SEM images captured at similar magnification validated the appearance of the apical epithelial surface in live DIC mode with respect to the macrotopology of cell junctions, the slight height differences between cells, and the distinctly patterned apical surface (compare Fig. 1K and L). At higher SEM magnification, we observed a richly microvilliated surface (Fig. 1M), with preserved binding of Salmonella to the host cells (Fig. 1N).

In conclusion, the combination of an upright water-dipping objective microscope and the novel AIC provide a system for simultaneous high-definition DIC imaging of both microbe and host cell features at the apical surface of human intestinal epithelial cell layers. This approach does not require the use of fluorescent dyes or labeled markers and allows for convenient semicorrelative SEM on samples fixed at the end of live-cell imaging.

### Resolving subsecond-scale microbial motility patterns along the apical surface of human intestinal epithelium.

Pathogenic gut microbes often use flagellar motility to reach and explore the epithelial surface. Motile pathogen behaviors have typically been studied atop artificial surfaces (e.g., plastic or glass) (3, 34) or occasionally under more physiological conditions (e.g., atop tissue explants [35]). However, under the latter conditions the imaging method relies on fluorescent reporters and high-intensity illumination that come at the price of phototoxicity and may alter the processes under study or do not permit simultaneous surface structure visualization. The AIC setup presented here should be ideally suited to study authentic pathogen motility patterns at the IEC surface under minimally perturbing conditions. To leverage this possibility, we explored the motility of two microbes atop IEC monolayers grown in AICs, the smaller bacterial pathogen Salmonella and the larger protozoan parasite Giardia.

**FIG 2:**
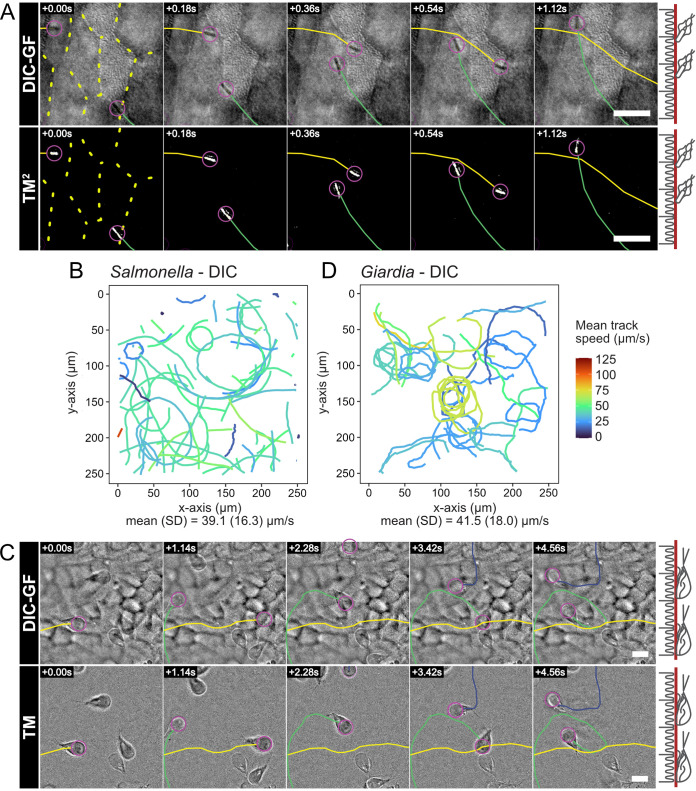
Tracking of microbes on IEC monolayers using DIC imaging resolves Salmonella and Giardia motility patterns. Confluent, differentiated IEC monolayers in AICs were infected with Salmonella*-mCherry* (A and B) or Giardia*-mNeonGreen* (C and D). The figure shows tracking from DIC time-lapse movies. Tracking using the respective fluorescent markers (for validation purposes) is shown in Fig. S2. DIC movies were processed using a Gaussian filter (DIC-GF) to remove uneven background illumination and achieve optimal contrast for Salmonella (A, top) and Giardia (C, top). Subsequently, the total DIC-GF stack temporal median projection was subtracted from every frame to specifically emphasize moving structures (TM), and pixel values were squared (TM^2^) to yield positive (white) pixels on a zero (black) background. The TM^2^ filtered time series was used to track all visible Salmonella using automated particle tracking (A, bottom). For Giardia, the TM filtered images were used for manual tracking of swimming trophozoites (C, bottom). A random sample of tracks within the field of view (FOV) was used to visualize Salmonella motility (B), while all manually tracked paths are shown for Giardia (D). The population means and standard deviations for the track speeds were calculated based on all available tracks (Fig. S2C and D). In panels A and C, a representation of the focal plane (red) is indicated on the right. Tracked microbes are indicated by magenta circles and tracks by continuous colored lines. Overlaid dashed lines indicate cell-cell junctions. Scale bars, 10 μm.

Peritrichous flagellum-driven Salmonella motility physically constrains stretches of the bacterial swim path atop surfaces, a phenomenon called near-surface swimming (NSS) (3). Using DIC imaging alone, we could successfully follow Salmonella NSS along the apical surface of the epithelium with high frame rates (up to ∼30 frames/s) at modest light intensity and with simultaneous visualization of apical epithelial topology (Fig. 2A, top row). Although bacterial NSS could easily be tracked manually based on the DIC images alone, greyscale images cannot be readily thresholded for automated downstream analysis by particle tracking software, as would be the norm for fluorescence imaging (Fig. S2A) (36). We therefore incorporated a squared temporal median (TM^2^) postprocessing filter (see Materials and Methods) to extract bacterial NSS information from the background of the relatively static apical IEC topology. The resulting filtered image stack enabled both segmentation of motile bacteria and automated particle tracking without the use of fluorescent markers (Fig. 2A, bottom row, and Movie S1). Automated tracking of Salmonella NSS in the TM^2^ filtered stack showed a variety of curved tracks with a mean speed of 39.1 μm/s (Fig. 2B, Fig. S2C) and mean turning angle of ∼14.68°/15 μm clockwise (Fig. S2E), corresponding to its flagellum’s counterclockwise spin (37, 38). Reassessment of this pattern by fluorescence imaging resulted in broadly similar tracks (Fig. S2A and C) but importantly did not allow simultaneous visualization of epithelial surface topology. These observations validate and extend previous studies of Salmonella motility (3, 35, 38–40) by mapping Salmonella NSS parameters atop a physiologically arranged epithelial surface and under minimally perturbing conditions.

10.1128/mbio.00022-22.3 FIG S2 Summary of tracked microbe dynamics (supplement to Fig. 2). Confluent, differentiated IEC monolayers were infected with Salmonella*-mCherry* or Giardia*-mNeonGreen*. Time series were acquired in the respective fluorescent channels, and a random sample of Salmonella tracks (A) and all Giardia tracks (B) within the field of view were used to visualize microbe motility. (C and D) The population mean and standard deviation for the track speeds were calculated based on all available tracks. (E and F) Imaging of the DIC channel for both microbes was used to calculate the turning angle over 15 μm. To remove angle change measurements from nonmotile Salmonella, only tracks with a mean track speed of >5 μm/s were included in the analysis. Since manual tracking of Giardia was only performed for motile trophozoites, these tracks were analyzed without filter. (E) The distribution of mean angle changes for Salmonella swimming tracks show a central tendency (red arrow) of the bacteria to continue a clockwise (+) rather than counterclockwise (−) circular pattern with small standard deviation (red area), which allows for more ellipsoid tracks and lateral movement. (F) For Giardia, the angle change distribution does not show a net clockwise or counterclockwise tendency but rather an almost straight mean trajectory with large standard deviation towards both directions. Crossbar plots indicate the median and interquartile range (IQR). Download FIG S2, TIF file, 1.0 MB. Copyright © 2022 van Rijn and Eriksson et al. 2022 van Rijn and Eriksson et al. https://creativecommons.org/licenses/by/4.0/ This content is distributed under the terms of the Creative Commons Attribution 4.0 International license.

10.1128/mbio.00022-22.6 MOVIE S1 Salmonella motility atop IEC monolayers (supplement to Fig. 2). The movie shows a real-time example of Salmonella motility on the apical surface of IEC monolayers cultured in an AIC. The bacteria and IEC apical surface are evident from the DIC channel (top), whereas TM filtering of the DIC time-lapse stack extracts information on moving bacteria (middle). For automated tracking of bacteria, the TM^2^ filtered stack provides a reliable representation of bacterial motility (bottom). Download Movie S1, AVI file, 19.3 MB. Copyright © 2022 van Rijn and Eriksson et al. 2022 van Rijn and Eriksson et al. https://creativecommons.org/licenses/by/4.0/ This content is distributed under the terms of the Creative Commons Attribution 4.0 International license.

Giardia trophozoites feature four pairs of flagella and use motility to swiftly approach the intestinal epithelium, followed by stable attachment using a ventral disk (30, 41). Giardia free-swimming motility in medium involves flexion of the caudal portion of the parasite body (34). On flat surfaces (i.e., glass), Giardia adapt the swimming mode to planar motility, largely driven by the flagella. Planar motility in this context is defined by a swimming pattern with the ventral disk continuously in the same plane as the attachment surface (34, 42). As these Giardia motility characteristics remain inferred from more simplistic experimental conditions (41), we performed live DIC imaging of early Giardia motility atop IEC monolayers within AICs. We again could simultaneously visualize the IEC surface and individual Giardia trophozoites and monitor parasite movements at a variety of frame rates (Fig. 2C, top row). The lower contrast and less predictable swim paths noted for these bigger protozoans compared to Salmonella were not well suited for automated particle tracking. However, an unsquared temporal median filtering (TM) step aided robust manual segmentation and tracking of Giardia motility (Fig. 2C, bottom row, and Movie S2). This revealed epithelium-proximal swimming in curved or circular tracks with a mean speed of 41.5 μm/s (Fig. 2D, Fig. S2D) and an almost straight mean turning angle of 0.41°/15 μm but with a large standard deviation in both the clockwise and counterclockwise directions (Fig. S2F). We validated these findings by fluorescence imaging of mNeonGreen-labeled Giardia and automated particle tracking (Fig. S2B and D). The speed values aligned well with the maximal speeds measured for Giardia during free swimming in media (up to ∼40 μm/s) (34). This indicates that when first approaching a polarized microvilliated epithelium, Giardia sustains maximal swim speed for a significant period after switching to planar 2D motility.

10.1128/mbio.00022-22.7 MOVIE S2 Giardia motility atop IEC monolayers (supplement to Fig. 2). The movie shows a real-time example of Giardia motility on the apical surface of IEC monolayers cultured in an AIC. Both the cellular surface topology and the attached or moving trophozoites are visible in the DIC channel (top), while moving Giardia are efficiently extracted using the TM filter (bottom). In cases where a trophozoite is motile in some frames of the movie but attached and stationary in most, the TM filter shows a shadow in the location of attachment. As is evident from this movie, the TM filter is especially useful to segment the individual flagella. Download Movie S2, AVI file, 19.4 MB. Copyright © 2022 van Rijn and Eriksson et al. 2022 van Rijn and Eriksson et al. https://creativecommons.org/licenses/by/4.0/ This content is distributed under the terms of the Creative Commons Attribution 4.0 International license.

Taken together, we demonstrate that DIC imaging in AICs provides a powerful solution for resolving microbial motility patterns atop a human intestinal epithelial cell layer. Specifically, this methodology simultaneously captures both apical IEC layer topology and single microbe behaviors at high frame rates, does not require fluorescent labeling, and, as such, avoids both potential problems with reporter toxicity/phototoxicity as well as the temporal delays that come with switching between multiple imaging channels. Moreover, the AIC technology will allow in-depth analysis of how microbial motility on the epithelial surface is impacted by physiologically relevant surface features (e.g., crevices formed at cell-cell junctions, extruding IECs, or overlaying mucus structures).

### Longer-term imaging reveals a previously unrecognized Salmonella Typhimurium infection cycle stage atop the epithelial surface.

Following NSS, Salmonella can adhere to the epithelial surface through a combination of transient interactions via dedicated adhesins and/or flagella and stable docking to the plasma membrane via type-III-secretion system-1 (TTSS-1) (43–46). Docking of the TTSS-1 tip subsequently permits the translocation of bacterial effector proteins into the host cell cytoplasm (1). A rich body of work in epithelial cell line models has shown that this leads to the near-instantaneous induction of large actin-dependent membrane ruffles and swift Salmonella invasion of the targeted cell (1). However, recent work has also shown that the invasion phenotype is dependent on the context of the host cell and suggests an influence of host cell polarization status (47, 48). It therefore remains less well understood how the physiological properties of intact nontransformed epithelia impact Salmonella infection cycle stage(s) at the host cell surface.

**FIG 3:**
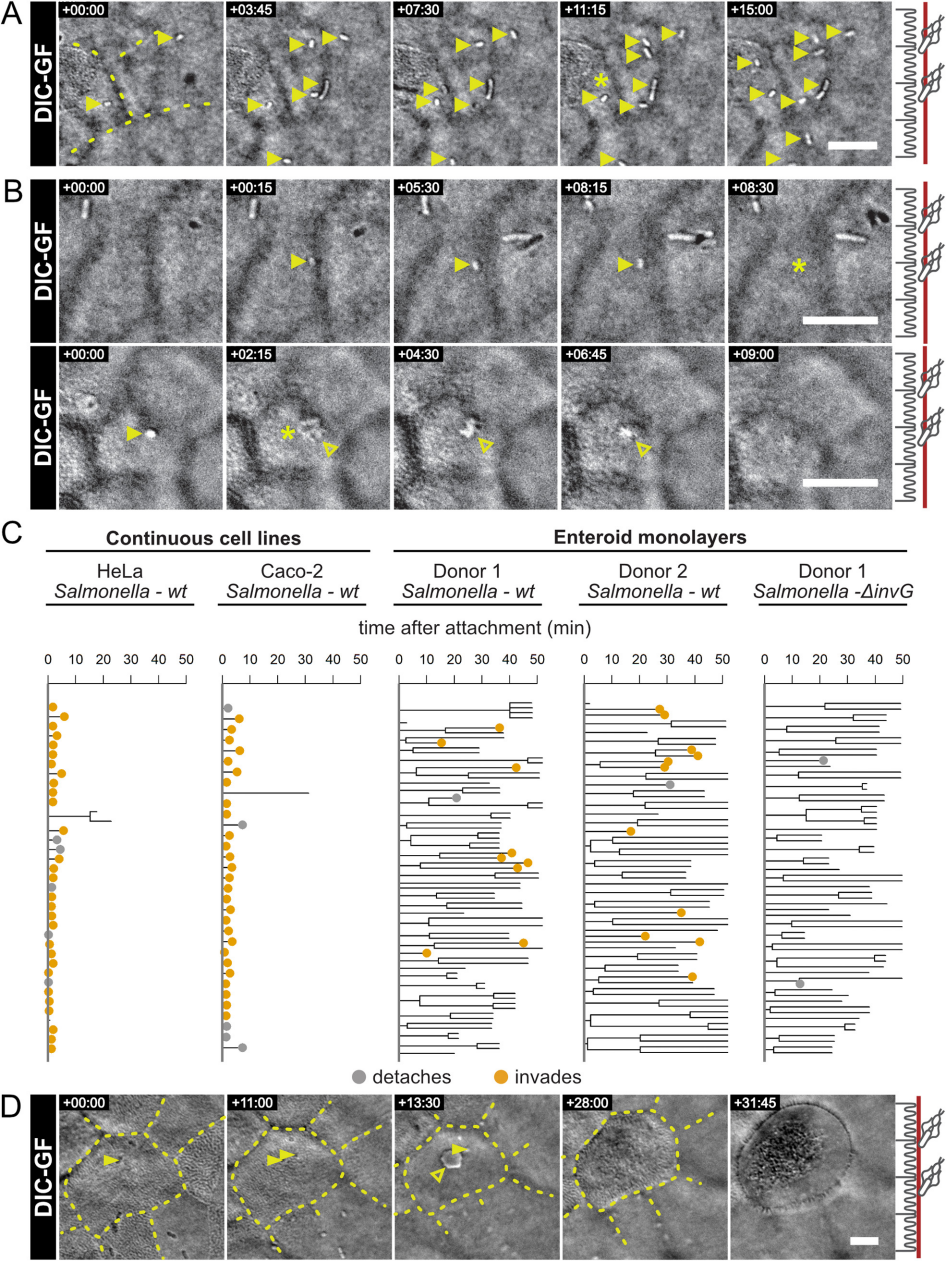
Salmonella infection cycle stages at the apical IEC surface. Differentiated IEC monolayers in AICs were infected with Salmonella and imaged every 15 s. (A) Salmonella was most commonly observed to attach to the apical surface for the duration of the experiment. A fraction of bacteria was unsuccessful in establishing a lasting adhesion, seen as a short attachment and sudden disappearance (asterisk). In other cases, Salmonella disappeared from the surface after prolonged attachment either in the absence of visible IEC surface perturbation (B, top row) or concomitant with the induction of phenotypically small and discreet (B, bottom row) host cell invasion structures. The various fates of Salmonella bacteria after initial attachment were quantified for infections of continuous cell lines (HeLa and Caco-2) and IEC monolayers from two independent enteroid lines (donor 1 and donor 2). The resulting surface behavior data sets are visualized as dendrograms, where each bacterium’s behavior is represented by a branch line through time. In these dendrograms, divisions are represented as branching points and detachment or invasion events are indicated by colored points. Line ends without an indicated point represent the end of the movie or loss of focus. (C) These dendrograms show a unique lingering behavior for Salmonella atop IEC monolayers that is not seen atop continuous cell lines (Fig. S3B and C). Infection of an IEC monolayer with the TTSS-1-deficient Salmonella *ΔinvG* strain showed that invasion is TTSS-1 dependent but surface lingering is not. (D) Invasion of host cells elicited prompt extrusion of some targeted IECs (Fig. S3D), which involved an inward movement of the surrounding IECs, evident from the movement of the cell-cell junctions. Throughout, a representation of the focal plane (red) is indicated on the right. Bacteria are indicated with solid yellow and invasion structures with hollow yellow arrowheads. Yellow asterisks indicate disappearing bacteria (detachment or invasion). Overlaid dashed lines indicate cell-cell junctions. Scale bars, 10 μm.

To survey the longer-term fate(s) of Salmonella on IECs, we performed 1-h infections of AIC-grown IEC monolayers from two independent donors and compared them to infections of HeLa and Caco-2 cells in the same setup. In the resulting movies, we observed an abundance of stationary bacteria on the surface of the IEC monolayers (Fig. 3A), which was not observed atop the continuous cell lines. The accumulation of these surface-attaching bacteria occurred in both donor cultures in a time-dependent manner (Fig. 3A), lasting until a given bacterium detached (Fig. 3B, top), invaded (Fig. 3B, bottom), or lingered atop the IEC surface until the end of the experiment. Strikingly, attachment most often (>75% of cases) did not lead to successful IEC invasion within 1 h postinfection (Fig. 3C, Fig. S3A and B), which was in stark contrast to the prompt invasion seen in both HeLa and Caco-2 infections (Fig. 3C, Fig. S3B and C). Nevertheless, IEC invasion through small and transient entry structures at the apical surface could still be detected in the AIC-grown IEC monolayers (Fig. 3B, bottom), albeit at much lower frequency than in the cell lines (Fig. 3B and C, Fig. S3B). Detachment of surface-associated bacteria also was seen only rarely (∼3% of cases), indicating that attachment/lingering on the IEC surface is generally quite stable (Fig. 3C, Fig. S3B). Indeed, Salmonella attachment to the IEC surface was stable enough to allow both daughter cells to remain attached after bacterial division (Fig. S3A). Notably, this surface attachment/lingering phenotype was independent of a functional TTSS-1, as the Salmonella *ΔinvG* TTSS-1-deficient strain (49) showed impaired invasion but not impaired attachment and lingering (Fig. 3C, Fig. S3B). Taken together, this prolonged lingering behavior of Salmonella atop IEC monolayers contrasts sharply with the observations in continuous cell line models. Specifically, attachment of Salmonella to the cell lines led to classical ruffle-mediated invasion (3, 47) already within 7 min post-attachment (Fig. S3C; exemplified in Movie S3). In contrast, the preinvasion lingering on IEC monolayers endured for at least ∼10 min with a median of ∼30 to 40 min within the 1-h experimental time frame (Fig. S3C).

10.1128/mbio.00022-22.4 FIG S3 Surface behavior of attached Salmonella (supplement to Fig. 3). Under the conditions reported in Fig. 3, attached bacteria lingered on the apical surface of IEC monolayers for long periods without the induction of entry structures. These attached bacteria could even be seen to grow and divide, where daughter cells dissociated and remained both attached to the IEC surface throughout the division cycle. (A) Images are shown in both the DIC and mCherry channels. (B) The surface behaviors of attached Salmonella bacteria in Fig. 3C were quantified and summarized to show the frequency of each event. (C) For those bacteria that successfully induced entry structure-mediated invasion, the time from attachment to invasion shows drastically longer lingering times on the surface of IEC monolayers versus continuous cell line cultures. (D) The numbers of extrusions were counted per field of view (FOV) for three replicate infections with Salmonella*-wt* or Δ*invG* strain, showing that the extrusion phenotype is indeed dependent on functional expression of the TTSS-1. Scale bars, 10 μm. Statistical significance of the number of extrusions/FOV/h was tested with a *t* test. *, *P < *0.05. Download FIG S3, TIF file, 1.7 MB. Copyright © 2022 van Rijn and Eriksson et al. 2022 van Rijn and Eriksson et al. https://creativecommons.org/licenses/by/4.0/ This content is distributed under the terms of the Creative Commons Attribution 4.0 International license.

10.1128/mbio.00022-22.8 MOVIE S3 Swift ruffle-mediated Salmonella invasion into HeLa cells. HeLa cells within AICs were infected with Salmonella and immediately imaged using the water-dipping objective microscope. Invasion of Salmonella into HeLa cells occurs through a prompt, extensive ruffling response on the side of the host cell, often induced within seconds to minutes after the bacterium contacts the plasma membrane. The attaching bacterium is indicated with a yellow arrow head. Time is indicated as minutes:seconds relative to the start of the movie. Download Movie S3, AVI file, 13.1 MB. Copyright © 2022 van Rijn and Eriksson et al. 2022 van Rijn and Eriksson et al. https://creativecommons.org/licenses/by/4.0/ This content is distributed under the terms of the Creative Commons Attribution 4.0 International license.

Successful entry of a bacterium was occasionally (3 to 6 events per hour per field of view) followed by prompt neighbor-coordinated extrusion of the entire targeted IEC from the monolayer (Fig. 3D, Fig. S3D, Movie S4). This response was not detectable in continuous cell line infections on this short time scale. However, the time frames and morphological appearance corresponded well with IEC extrusions previously reported in Salmonella-infected 3D enteroids (15) and *in vivo* in mice and calves (50–52). Therefore, fast infected host cell extrusions appear to be a property of nontransformed intestinal epithelia, which is well recapitulated in the current setup.

**FIG 4:**
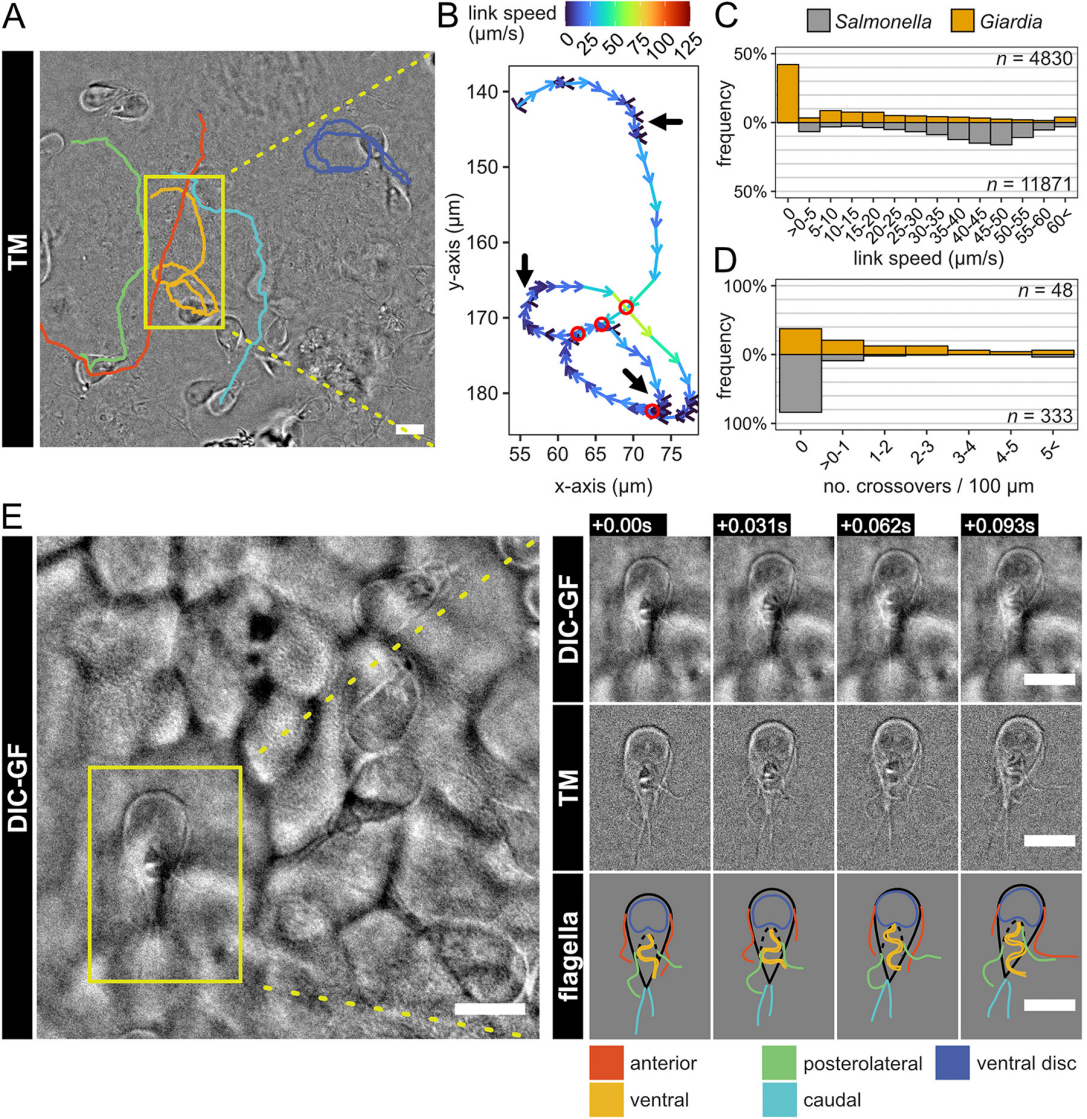
Giardia trophozoite exploration of the IEC surface. (A) The link speeds of manually segmented Giardia swimming tracks were analyzed (Fig. S4) and representative tracks showing both straight swimming and the common circular pattern were plotted on the DIC overview image. (B) Upon inspection of link speeds, the trophozoites were found to swim with highly variable speeds within a single track, with intermittent pauses upon sections of the epithelium defined by a link speed of 0 μm/s (black arrows). (C) Quantification within all Giardia and Salmonella tracks of >100-μm length emphasized this stopping behavior of Giardia, while Salmonella bacteria were seen to swim at a more constant speed, rarely approaching link speeds of 0 μm/s (Fig. S4). Giardia circling behavior within these tracks, which results in revisiting of the same area, was measured by identifying intratrack crossover points (red circles in panel B). (D) The resulting metric indicated that 62% of Giardia tracks revisit a spot in the track versus only 16% for Salmonella, represented by >0 crossovers/100 μm/FOV (Fig. S4). (E) Upon closer inspection of a single, attached trophozoite, the DIC-GF channel showed movement of individual flagellar pairs (top row), which could be manually segmented using the TM filtered image to monitor their shape and position over time (middle and bottom panels to the right, respectively). Scale bars, 10 μm.

10.1128/mbio.00022-22.5 FIG S4 Summary of link speeds and crossover frequencies for Giardia and Salmonella swim tracks atop the IEC surface (supplement to Fig. 4C). The individual, manually tracked paths of Giardia trophozoite DIC imaging were used to analyze the distribution of link speeds (A, top). Salmonella tracks of similar lengths were used for comparison (A, bottom). Shown are scatter plots of link speeds within a random sample of 25 individual tracks for each pathogen. Most Giardia tracks show a significant number of links with a link speed of 0 μm/s, indicating that the trophozoites regularly come to a full stop within one track. The grey zone indicates the IQR for all shown tracks, and the black line indicates the median link speed for all shown tracks. Below is a summary of descriptive parameters for the link speed (B) and crossover (C) analysis shown in Fig. 4C and D. Download FIG S4, TIF file, 0.7 MB. Copyright © 2022 van Rijn and Eriksson et al. 2022 van Rijn and Eriksson et al. https://creativecommons.org/licenses/by/4.0/ This content is distributed under the terms of the Creative Commons Attribution 4.0 International license.

In conclusion, our results show that imaging of epithelial infection within AICs opens up new avenues for the microscope-aided study of bacterial behavior at the apical border of a human IEC layer, compatible with both short (subsecond) and long (hours or more) time scales. By employing this technology, we find that Salmonella organisms do not invade a physiologically arranged human IEC layer in the same quick manner as they do in tumor-derived cell line models. Instead, stable and prolonged bacterial colonization of the IEC surface constitutes a significant infection cycle stage, which transitions into productive IEC invasion for <25% of attached bacteria within the first hour of infection. The molecular and physiological basis, and *in vivo* significance, of these observations constitutes an intriguing area for future research.

### Giardia alternates between rapid swimming and intermittent attachment during circular local surface exploration.

Earlier reports of Giardia behavior on glass have described the trophozoites’ preattachment swimming pattern as circular movements, largely driven by beating of the anterior and ventral flagella and steered by lateral bending of the caudal region (34, 42). Although the eventual attachment of Giardia has been validated in numerous reports (41, 53) and also in epithelial cell line cultures (5, 54), exploratory trophozoite behavior that leads to successful stable attachment has not yet been studied atop an intact epithelial surface.

Therefore, we homed in on individual Giardia swim tracks in IEC layer regions that harbored both moving and stably adhered trophozoites. The subset of motile Giardia was observed to swim in circular tracks, which gradually shifted their position on top of the monolayer (Fig. 4A). From these tracks, the link speed was calculated as the displacement divided by the time interval between two subsequent frames (Fig. 4B). By this analysis, it became apparent that Giardia trophozoites typically swim at the epithelial surface in a repetitive stopping pattern, featuring one or multiple intermittent stops within a given track (defined by a link speed of 0 μm/s) (Fig. 4B, black arrows). This behavior resulted in a highly variable intratrack link speed, which was not seen for the more constantly moving Salmonella bacteria in tracks of similar lengths (Fig. 4C, Fig. S4A and B). Furthermore, surface-scanning Giardia often engaged in periods of repetitive circling atop smaller regions of the epithelium (Fig. 4B). As a measure for this circling behavior, we extracted the number of within-track crossover events for individual trophozoites (Fig. 4B, red circles). We found that 62% of motile Giardia tracks >100 μm in length included at least one crossover event, whereas only 16% of similarly long tracks did so for Salmonella (Fig. 4D, Fig. S4C). Therefore, our data indicate that Giardia trophozoites employ localized repetitive circling and intermittent attachments to explore the IEC surface. It seems plausible that this behavior maximizes the parasite’s ability to find ideal permanent attachment sites within an epithelial region.

With four pairs of flagella and an adhesive ventral disk, the behavior of Giardia trophozoites at a surface is the result of a complicated interplay of propulsion and adhesive forces. Aside from their role in propulsion, flagellar movement has been described to influence correct positioning of the adhesive disk on the attachment surface, although the exact mechanism of that process remains a topic of discussion (34, 53). To test if our AIC setup would allow studies of individual flagellar movements within the context of epithelial infection, we imaged single Giardia cells atop the IEC monolayer at high frame rates. We found that DIC imaging alone was indeed sufficient to distinguish the movement of individual flagella on intermittently attached trophozoites (Fig. 4E, left and top row). Application of the TM filter further facilitated manual segmentation of individual flagella (Fig. 4E, middle row, and Movie S2), and the imaging resolution was sufficient to indicate the movement of all four pairs of flagella over time (Fig. 4E, middle and bottom row). This allowed us to determine that all of the anterior, posterolateral, and ventral flagella exhibit continued movement in intermittently attached Giardia on IEC monolayers. Consequently, intermittent Giardia stopping at the IEC surface is not caused by the temporary cessation of flagellar beating. This finding also illustrates the power of the AIC technology to resolve dynamic host-pathogen interactions with a close to physiologically arranged epithelial surface at subcellular resolution.

## DISCUSSION

Recent work has shown that intestinal organoid-derived monolayers grown on permeable supports provide a physiologically relevant culture model for host-pathogen interaction, applicable to bacteria (21–26, 28), parasites (27), and viruses (20). In this context, organoids bridge the gap between the complexity and polarized nature of the epithelium encountered *in vivo* and the convenient (yet sometimes misrepresentative) properties of continuous cultured cell lines. Furthermore, 2D organoid cultures offer an attractive alternative to 3D culture specifically for studies of microbe interactions with the apical host cell surface. The latter can be microinjected (15, 55, 56), fragmented (57), or inverted (58), but because of the 3D topology, these structures are better suited for population-scale dynamics than single-microbe behavior in a stable apical plane. While live-cell imaging of microbe dynamics has been published on monolayers of continuous cell lines (3, 59, 60) and, to an extent, in microinjected 3D enteroids (15), dynamic behaviors of microbes at the surface of a physiologically arranged human intestinal epithelium remain poorly investigated. We show that this shortcoming can be explained by the inherent conflict between the technical prerequisites for high-definition light microscopy (e.g., a short working distance, a thin specimen, and an optically inert substrate) and the current best practices for establishment of polarized IEC layer cultures.

Therefore, here we developed a custom imaging chamber containing a coated alumina membrane substrate. This AIC is compatible with water-dipping objective imaging to maximize optical resolution, while the alumina membrane has optical properties superior to permeable plastic cell culture supports. With these improvements to the ASC-derived IEC monolayer culture system, we present an optimized method for high-definition imaging of host-microbe interactions at the apical surface of confluent, polarized, nontransformed, human gut epithelia. Finally, we used this technique to uncover previously unknown behaviors of both Salmonella and Giardia on IEC monolayers.

Our AIC allows tracing of the behavior of individual Salmonella cells over time from the moment they come in proximity to the apical cell surface. Previous work using continuous cell lines and *in vivo* infection models have shown that flagellum-dependent Salmonella motility drives the approach toward host cells (39, 61) and initiates subsequent NSS behavior in search of suitable entry sites (3, 35). Our findings regarding Salmonella’s curved NSS atop IEC monolayers (Fig. 2A and B, Fig. S2A, C, and E) agree with this model of approach. Upon contact with the host epithelium, Salmonella can bind to the host cells using a combination of adhesins and the TTSS-1 (43, 62). However, in contrast to infections of HeLa and Caco-2 cells, we found that successful binding to the IEC monolayer does not lead to prompt ruffle-mediated invasion (Fig. 3A to C). Rather, we observed that bacterial attachment is not dependent on the TTSS-1 (Fig. 3C), that invasion upon attachment is less frequent in IEC monolayers (<25%) than in continuous cell lines (>75%) (Fig. S3B), and that Salmonella often lingered atop the epithelial surface without invading within an hour postinfection (Fig. 3C). This prolonged bacterial attachment, verified in two independent enteroid lines, suggests an additional biologically relevant epithelial colonization stage in the infection cycle of Salmonella, in apparent analogy to attaching-effacing enteropathogens that use the epithelial surface as a primary colonization niche (63). A contribution of the dense apical glycocalyx atop IECs to this Salmonella lingering phenotype also appears plausible (64).

Nevertheless, Salmonella can still invade IECs (Fig. 3B), albeit at lower frequency and through entry structures that were smaller and more transient than those seen in infections of flat-growing cell lines (Fig. 3B, Movie S3) (47). This is broadly in line with the more discreet mode of Salmonella TTSS-1-dependent IEC invasion described in the intact mouse intestine (47). In agreement with existing literature (17, 50–52, 65–68), we also observed that successful invasion of the IEC monolayer can lead to immediate extrusion of the entire infected cell (Fig. 3D). However, the time from invasion to extrusion seems at times to vary between cells within the same field of view. The molecular obstacle(s) that makes polarized nontransformed IEC layers more challenging to invade than typical cell lines, and the specific mechanisms that determine the acuteness of host cell extrusions, constitute intriguing areas for further inquiry.

Previous studies of Giardia infections have shown that the motile trophozoites can attach to both inert substrates like glass (41) and to microvilliated cell surfaces *in vivo* (69) and in culture (5). Research on interaction dynamics of Giardia with inert substrates has shown that flagellar motility is essential for motility, cell division, and selection of suitable attachment sites (34, 42, 53, 70, 71), although this behavior has not yet been studied in real time on live cells. Here, we report, to our knowledge, the first study of Giardia swimming on top of cultured cells and on polarized IEC monolayers at that. In general agreement with earlier studies on glass (34), we found that Giardia exhibits circular, planar swimming above the attachment surface. Specifically, we found that Giardia planar swimming speeds average around 30 to 40 μm/s with short bursts of up to ∼150 μm/s (Fig. S4, Fig. S4B). These speeds are much higher than those of the preattachment swimming that has been characterized before on glass (34, 42, 71–73). Furthermore, we see repetitive circular swimming (Fig. 4B and D) interceded by short stretches of straight swimming (Fig. 4A). Finally, we show that trophozoite planar swimming along the epithelial surface cooccurs with intermittent, transient attachments to the host cells (Fig. 4B and C). Therefore, our results suggest that Giardia planar swimming is geared toward a local surface-scanning pattern, optimized to select a suitable attachment site within a given region. The circular swimming pattern could aid in this process by repeatedly visiting promising sites of adhesion until an unknown threshold of stable adhesion is reached.

Earlier work on glass has elegantly shown that flagellar beating is instrumental to the planar swimming behavior of Giardia, although the respective contribution of the anterior, posterolateral, and ventral flagellar pair is still a matter of debate (34, 42). Strikingly, temporal median filtering in our study resolved the continuous movements of anterior, posterolateral, and ventral flagella in intermittently surface-attached Giardia (Fig. 4E). Although we cannot directly measure attachment force, this suggests that the transient Giardia pauses during surface search can be explained by ventral disk or ventrolateral flange (69, 74) engagement with the surface rather than by an on-off behavior of flagellar propulsion. As the AIC can be used to study both the overall swim patterns and the movement of individual flagella, this technique is ideally suited to characterize the complete spectrum of Giardia (and other parasites) behaviors atop intestinal epithelia.

The findings described above highlight the power of the AIC approach to study microbial interactions at the apical border of a polarized nontransformed IEC layer. Still, the current system does not represent the full complexity of the *in vivo* infection environment. There, the invading microbe will be confronted with competing microbiota in a partially or fully anaerobic environment, subjected to peristalsis of the gut and flow of luminal content and variation of these parameters along the proximal-distal axis of the human intestine. The intestine also presents a highly diverse barrier of epithelial cell subtypes, where, e.g., goblet cells produce a protective mucus barrier, Paneth cells produce a cocktail of antimicrobial peptides, microfold (M) cells continuously sample the luminal content, and the exact cell type composition varies along both the proximal-distal and the crypt-villus axis of the epithelium (75). All these influences contribute to the clinical manifestations of human infections, with Salmonella typically colonizing the distal ileum and proximal colon, while Giardia colonizes the proximal small intestine. Our current observations are limited to jejunum-derived enterocyte-enriched monolayers. However, enteroids/colonoids retain their location-specific expression profiles (76) and can be induced to differentiate into the various IEC subsets (75), and there is precedence for exogenous introduction of other key niche features, such as anaerobic conditions and select microbiota members (26). Based on this, further adaptation of the AIC imaging technology should permit systematic studies of epithelial infection dynamics across the diversity of gut segments, IEC differentiation stages, and niche-specific traits.

## MATERIALS AND METHODS

### Ethics statement.

Human jejunal ASC-derived enteroids from two independent donors were generated from resected tissue acquired from routine bariatric surgery following prior informed consent. All personal information was pseudonymized, and the patients’ identities were unknown to researchers working with the tissue samples. The procedures were approved by the local governing body (Etikprövningsmyndigheten, Uppsala, Sweden) under license number 2010-157 with addenda 2010-157-1 (2018-06-13) and 2020–05754 (2020-10-26)

### Apical imaging chambers.

The AICs for 13-mm alumina Whatman Anodisc membranes with 0.2-μm pores (GE Healthcare, Little Chalfont, United Kingdom) were designed in-house using FreeCAD v0.19 (https://www.freecadweb.org). The chamber comprises a bottom holder and top cover, which fit together using a friction press fit to hold the alumina membrane in place, with culture medium compartments generated on each side of the membrane. The top cover contains a groove to hold a silicone gasket (product number 527–9790; RS Components Ltd., Corby, United Kingdom) with inner diameter of 7.65 mm in place. AIC design files are available for noncommercial use at https://doi.org/10.17044/scilifelab.16402539. The AIC designs were printed using an Original Prusa MINI+ (Prusa Research, Prague, Czech Republic) 3D printer with a 0.4-mm nozzle and a layer height of 0.2 mm. The AICs were printed in 1.75 mm, clear, natural PLA filament (832-0210; RS Components Ltd.).

### Enteroid culture.

Human jejunal enteroid cultures (pseudonym IDs 18-8 and 18-9) were established and kept as described previously (15, 17). Briefly, pieces of intestinal resections were washed thoroughly in ice-cold phosphate-buffered saline (PBS), and epithelial crypts were subsequently dissociated using gentle cell dissociation reagent (STEMCELL Technologies, Vancouver, BC, Canada) by nutating at 4°C for 30 min, followed by trituration. The resulting epithelial fragments were filtered through a 70-μm cell strainer and crypt-enriched fractions suspended in 50 μl Matrigel (Corning, Corning, NY, USA) domes in a 24-well plate. The embedded crypts were cultured in OGM (IntestiCult organoid growth medium [Human]; STEMCELL Technologies) with 100 U/ml penicillin-streptomycin (Thermo Fisher [Gibco], Waltham, MA, USA) at 37°C and 5% CO_2_. Growth medium was refreshed every 2 to 3 days.

For maintenance of human enteroids, the structures were passaged weekly at a ratio of circa 1:8 by mechanical dissociation. The Matrigel domes were manually broken up by pipetting with gentle cell dissociation reagent and then washed once with Dulbecco’s modified Eagle’s medium (DMEM)-F12–1.25% bovine serum albumin (BSA). The resulting suspended enteroids were disrupted by triturating 15 to 20 times with a 200-μl pipette tip. Following disruption, enteroid fragments were again suspended in 50 μl Matrigel-Intesticult at a ratio of 3:1, divided over 3 domes per well in a 24-well plate, and cultured at 37°C and 5% CO_2_.

### Enteroid-derived IEC monolayer culture.

Human IEC monolayers were cultured on either 24-well transparent polyethylene terephthalate (PET) tissue culture inserts with 0.4-μm pores (Sarstedt, Nümbrecht, Germany) or 13-mm-diameter alumina Whatman Anodisc membranes. The PET transwell inserts were coated with 40× diluted Matrigel in PBS for 1 h at room temperature prior to use. After this time, the coating was completely removed and the cell suspension was immediately added to the transwell inserts. The alumina membranes were coated with extracellular matrix as well but required more extensive pretreatment. First, the alumina membranes were soaked in 30% H_2_O_2_ for 1 h at room temperature to add negatively charged hydroxyl groups to the surface (32) and allow protein binding. Next, the alumina membranes were washed in sterile distilled water (dH_2_O) and incubated in 0.1 mg/ml poly-l-lysine (Sigma-Aldrich, Stockholm, Sweden) in dH_2_O for 5 min to prepare the surface for Matrigel coating. After poly-l-lysine coating, the membranes were air dried in a laminar-flow cabinet for ∼2 h or overnight. Finally, the alumina membranes were soaked in 40× diluted Matrigel in dH_2_O for 1 h and air dried again. After coating, the membranes were mounted within AICs.

Human enteroids were dissociated into single-cell suspensions as described before (17). Briefly, circa one well of enteroids per membrane was dissociated into single cells at day 7 after passaging. The enteroids were first taken up from the Matrigel in gentle dissociation reagent and then washed in PBS–1.25% BSA and dissociated into single cells using TrypLE Express (Thermo Fisher [Gibco]) for 5 to 10 min at 37°C. Cells were then spun down at 300 relative centrifugal force for 5 min and resuspended in OGM+Y (Rho kinase inhibitor Y-27632 [10 μM]; Sigma). Finally, the cells were counted manually, and 3.0 × 10^5^ cells were seeded into the apical compartment of PET transwells in 150 μl (600 μl medium in the bottom compartment, 24-well plate wells) or into the apical compartment of AICs in 75 μl (600 μl medium in the bottom compartment, 12-well plate wells). The monolayers typically grew confluent in 2 to 4 days, after which the cells were differentiated toward an enterocyte phenotype by deprivation of WNT signaling for 4 to 5 days. The medium for differentiation consisted of DMEM-F12 supplemented with 5% R-Spondin1 conditioned medium (home made from Cultrex 293T R-spondin1-expressing cells; R&D Systems, MN, USA), 10% Noggin conditioned medium (home made with HEK293-mNoggin-Fc cells; kindly provided by Hans Clevers, Utrecht University), 50 ng/ml mouse recombinant EGF (Sigma-Aldrich), 1× B27 supplement (Gibco), 1.25 mM *N*-acetyl cysteine, and 100 U/ml penicillin-streptomycin (9, 10).

### IEC substrate attachment assay.

IEC monolayers were grown as described above on alumina membranes coated with PLL alone, H_2_O_2_ and PLL, or H_2_O_2_ plus PLL plus MG. After 3 days of growth in OGM+Y, the monolayers were fixed in 4% paraformaldehyde and stained for nuclei with 4′,6-diamidino-2-phenylindole (DAPI). Imaging was performed for DIC and DAPI fluorescence on a custom-built microscope based on an Eclipse Ti2 body (Nikon), using a 4×/0.2-numeric-aperture (NA) Plan Apo Lambda air objective (Nikon) and a backlit sCMOS camera with a pixel size of 2.8 μm (Prime 95B; Photometrics). Fluorescence was excited using a Spectra-X light engine (Lumencor) and emission was collected through a quadruple bandpass filter (89402; Chroma).

### Salmonella Typhimurium strains, plasmid, culture, and infection.

All Salmonella infections in this study were performed with Salmonella enterica serovar Typhimurium, SL1344 (SB300) (77), as the wild type. Salmonella *ΔinvG* strain (49) was used as a noninvasive strain. For validation by standard fluorescence microscopy, the strains carried a pFPV-mCherry (rpsM-mCherry; Addgene plasmid number 20956) plasmid directing constitutive mCherry expression (78). As reported previously, expression of this mCherry construct did not influence motility or invasive behavior of Salmonella SL1344. For IEC monolayer infections, Salmonella inoculi were grown in LB, 0.3 M NaCl (Sigma-Aldrich) for 12 h overnight, with 50 μg/ml ampicillin in cases of plasmid-carrying strains. The following day, a 1:20 dilution was subcultured in LB, 0.3 M NaCl without antibiotics for 4 h. For subsequent infection of monolayer cultures, the 4-h inoculum was diluted to 1.0 × 10^8^ CFU/ml in DMEM-F12 (Thermo Fisher [Gibco]) without antibiotics, of which 10 μl was used for each infection, resulting in 1.0 × 10^6^ CFU per monolayer.

### Giardia intestinalis culture and infection.

Giardia intestinalis isolate WB, clone C6 (ATCC 30957), was used in this study. For validation by standard fluorescence microscopy, a modified Giardia line constitutively expressing mNeonGreen was generated (described below). Giardia trophozoites were grown at 37°C in 10-ml flat plastic tubes (Thermo Fisher Nunc, MA, USA) or 50-ml tubes (Sarstedt, Germany) filled with TYDK medium (also known as modified TYI-S-33 or Keister's medium) (79), supplemented with 10% heat-inactivated bovine serum (Gibco, Thermo Fisher MA, United States). All materials used in the TYDK medium were purchased from Sigma-Aldrich (MO, USA) unless otherwise stated. For IEC monolayer infections, Giardia trophozoites were grown until approximately 70% confluence and washed once with TYDK to remove dead cells. Further, trophozoites were incubated on ice (12 min), counted, and pelleted by centrifugation (800 × *g*, 10 min, 4°C). Cells were washed once in 1 ml DMEM-F12 (Thermo Fisher [Gibco]), centrifuged, and diluted to 2 × 10^7^ or 4 × 10^7^ trophozoites/ml using DMEM-F12, of which 10 μl was used for infection, resulting in 2 × 10^5^ to 4 × 10^5^ trophozoites per monolayer.

### mNeonGreen plasmid construction and Giardia trophozoite transfection.

To visualize Giardia trophozoites on IEC monolayers by fluorescence microscopy, we created a trophozoite strain constitutively expressing mNeonGreen under the control of the beta-giardin promoter. The mNeonGreen gene was PCR amplified from the pNCS-mNeonGreen plasmid (Allele Biotechnology, CA, USA) and the beta-giardin 5′- and 3′-untranslated regions were amplified from genomic DNA of the WB isolate (see Table S1 in the supplemental material). The PCR fragments were fused by overlap extension PCR and cloned into the integration vector pPacV-Integ-HA-C (80) using XbaI/PacI restriction sites. Giardia trophozoites were electroporated as previously described (81). Transgenic parasites that had the mNeonGreen expression cassette integrated on the chromosome were selected by adding puromycin (50 μg/ml) to the culture medium approximately 16 h after transfection. To ensure homogeneous mNeonGreen expression in the culture, we created a clonal trophozoite population from the original mNeonGreen stable transfectant population using serial dilution. Briefly, the original trophozoite culture was diluted in TYDK and seeded as single cells into the wells of a 96-well plate. Wells reaching 70 to 80% confluence were selected and grown in 10-ml tubes containing TYDK. After the establishment of the clonal mNeonGreen strains, the trophozoites were grown without antibiotics.

10.1128/mbio.00022-22.1 TABLE S1 Sequences of the primers used for the construction of the Giardia*-mNeonGreen* line. Download Table S1, PDF file, 0.3 MB. Copyright © 2022 van Rijn and Eriksson et al. 2022 van Rijn and Eriksson et al. https://creativecommons.org/licenses/by/4.0/ This content is distributed under the terms of the Creative Commons Attribution 4.0 International license.

### Fixed IEC monolayer imaging.

Differentiated IEC monolayers grown in AICs were fixed in 4% paraformaldehyde for 30 min and permeabilized with 0.1% Triton X-100 for 10 min. The cells then were stained with phalloidin-AF488 (Thermo Fisher) and DAPI (Sigma-Aldrich) counterstain for 30 min in PBS. Subsequently, the alumina membrane with cells was removed from the plastic membrane holder, washed, and mounted under a 0.17-μm coverslip in Mowiol 4-88 (Sigma-Aldrich). The samples were imaged on a Zeiss LSM700 inverted point-scanning microscope system with a 63×/1.4 NA oil immersion objective using a voxel size of 70.6 nm (*x* and *y*) and 0.54 μm (*z*-sections).

### Live-cell infection imaging.

Live-cell imaging was performed on an inverted Nikon Ti-eclipse microscope (Nikon Corporation, Tokyo, Japan) with a 60×/1.4 NA Nikon PLAN APO objective (0.19-mm working distance [WD]), Nikon condenser (0.52 NA, long working distance [LWD]), and Andor Zyla sCMOS camera (Abingdon, Oxfordshire, UK) with pixel size of 108 nm for Fig. 1C, D, and F to I and a custom upright microscope for all other experiments. The upright microscope is a custom build, based on the Thorlabs Cerna upright microscopy system (Newton, NJ, USA), with a heated 60×/1.0 NA Nikon CFI APO NIR objective (2.8 mm WD) and a Nikon d-CUO DIC oil condenser (1.4 NA) controlled by Micro-Manager 2.0-gamma (82). Images were acquired with an ORCA-Fusion camera (model number C14440-20UP; Hamamatsu Photonics, Hamamatsu City, Japan), with a final pixel size of 109 nm. Transmitted light was supplied by a 530-nm Thorlabs LED (M530L3) to minimize phototoxicity and chromatic aberrations. The microscope chamber was maintained at 37°C in a moisturized 5% CO_2_ atmosphere, and an objective heater was used. Samples in AICs were placed in 35-mm glass-bottom dishes (Cellvis, Mountain View, CA, USA) in 3 ml DMEM-F12 without antibiotics in the microscope’s light path and allowed to equilibrate for 30 min. Salmonella or Giardia then was added in premade dilutions directly underneath the objective, and imaging was started immediately, with <20-ms exposure times for DIC imaging and <50 ms for fluorescence imaging.

### HeLa and Caco-2 cell line infections.

HeLa CCL-2 cells (ATCC) were maintained in DMEM (Gibco) with 10% fetal calf serum (FCS) (Gibco) and 100 U/ml penicillin-streptomycin. Human colon adenocarcinoma cells (Caco-2), clone TC7 (83), were maintained in DMEM with 10% FCS, MEM nonessential amino acids solution (Gibco), and 100 U/ml penicillin-streptomycin. Both cell lines were cultured in plastic culture flasks and passaged every 2 to 3 days. For infection, 4.2 × 10^4^/cm^2^ HeLa cells or 2.1 × 10^5^/cm^2^ Caco-2 cells were seeded in AICs or on 35-mm glass-bottom dishes (Cellvis, Mountain View, CA, USA). Infection was performed the next day with 1.0 × 10^7^ CFU Salmonella in DMEM. Imaging was performed in the upright water-dipping microscope described above.

### SEM imaging.

For SEM analysis of AIC-grown monolayers, Salmonella-infected IEC monolayers were fixed at 40 min postinfection and compared with uninfected controls. To fix the samples, the monolayers were gently washed once with PBS and fixed at 4°C overnight with 2.5% glutaraldehyde (Sigma) in 0.1 M PHEM buffer [60 mM piperazine-*N*,*N*′-bis(2-ethanesulfonic acid), 25 mM HEPES, 10 mM EGTA, and 4 mM MgSO_4_·7H_2_0, pH 6.9]. Prior to SEM imaging, the samples were dehydrated in series of graded ethanol, critical point dried (Leica EM CPD300), and coated with 5-nm platinum (Quorum Q150T-ES sputter coater). The sample morphology was examined by a field emission scanning electron microscope (FESEM; Carl Zeiss Merlin) using in-lens and in-chamber secondary electron detectors at accelerating voltage of 4 kV and probe current of 100 pA.

### Image processing.

The acquired microscopy images were processed with Fiji (84). DIC images in Fig. 2 to 4 and Fig. S3 were filtered to acquire an even field of illumination by subtracting a (30-pixel sigma) Gaussian blurred projection from the original. Where indicated, a temporal median (TM) filter was used to extract quickly moving structures (e.g., motile Salmonella, Giardia, and moving flagella) by subtracting the median projection of the time stack from each frame. To enable automated particle tracking of moving Salmonella in DIC time series, pixel values of the signed 32-bit TM filtered stacks were squared to convert both dark and light bacteria in the TM-filtered image to positive values. These TM^2^-filtered images were used for automated particle tracking. Both automated and manual particle tracking was performed with Trackmate for ImageJ v6.0.1 (36).

### Tracking analysis and statistics.

Data analysis was performed with R (85) and RStudio (86) and packages available from the Comprehensive R Archive Network (CRAN; https://cran.r-project.org/). Tracking statistics were exported from Trackmate, and actual frame intervals were obtained from the image metadata using the Cellocity python package (87) to correct for variations between frames, which would be reflected in inaccurate link speeds. Subsequently, the track data were formatted and plotted in R with the tidyverse (88) package. Tracks with a mean speed of >100 μm/s were manually confirmed to be false positives of automated tracking and therefore were excluded from analysis. Track angle changes for DIC-tracked swimming paths were calculated using functions based on the trajr package (89) with an interval of three links. For the link speed and crossover analyses (Fig. 4C and D, Fig. S4), only tracks with a minimum track length of 100 μm were included in the analysis. The three-link angle change was divided by the distance travelled from the reference link to calculate the angle change per micron. To aid interpretation of this parameter, the angle change/micron was normalized to angle change/15 μm, the average distance travelled in 3 links. Crossovers for each link in each track were counted by a R-implementation of the “Intersection of two lines in three-space” algorithm described in reference 90. Other supporting packages for R include ggpubr, rstatix, ggbeeswarm, viridis, and scales.

10.1128/mbio.00022-22.9 MOVIE S4 Salmonella invasion into IEC monolayers leads to rapid host cell extrusion. An AIC-grown IEC monolayer was infected with Salmonella and imaged using the water-dipping objective microscope. A bacterium can be seen to attach to the apical cell surface, where it subsequently divides. One daughter cell then induces a relatively large (compare to the small entry structure in Fig. 3B), transient entry structure. Upon invasion of the host cell, the epithelium responds with a prompt extrusion of the entire infected cell. Time is indicated as minutes:seconds relative to the start of the movie. The bacteria of interest are indicated with yellow arrow heads. Download Movie S4, AVI file, 10.7 MB. Copyright © 2022 van Rijn and Eriksson et al. 2022 van Rijn and Eriksson et al. https://creativecommons.org/licenses/by/4.0/ This content is distributed under the terms of the Creative Commons Attribution 4.0 International license.
