# Supplementary figures and images for: High-Definition DIC Imaging Uncovers Transient Stages of Pathogen Infection Cycles on the Surface of Human Adult Stem Cell-Derived Intestinal Epithelium
Source: mBio. 2022 Feb 1;13(1):e00022-22. doi: 10.1128/mbio.00022-22 (PMC8805028; doi:10.1128/mbio.00022-22)

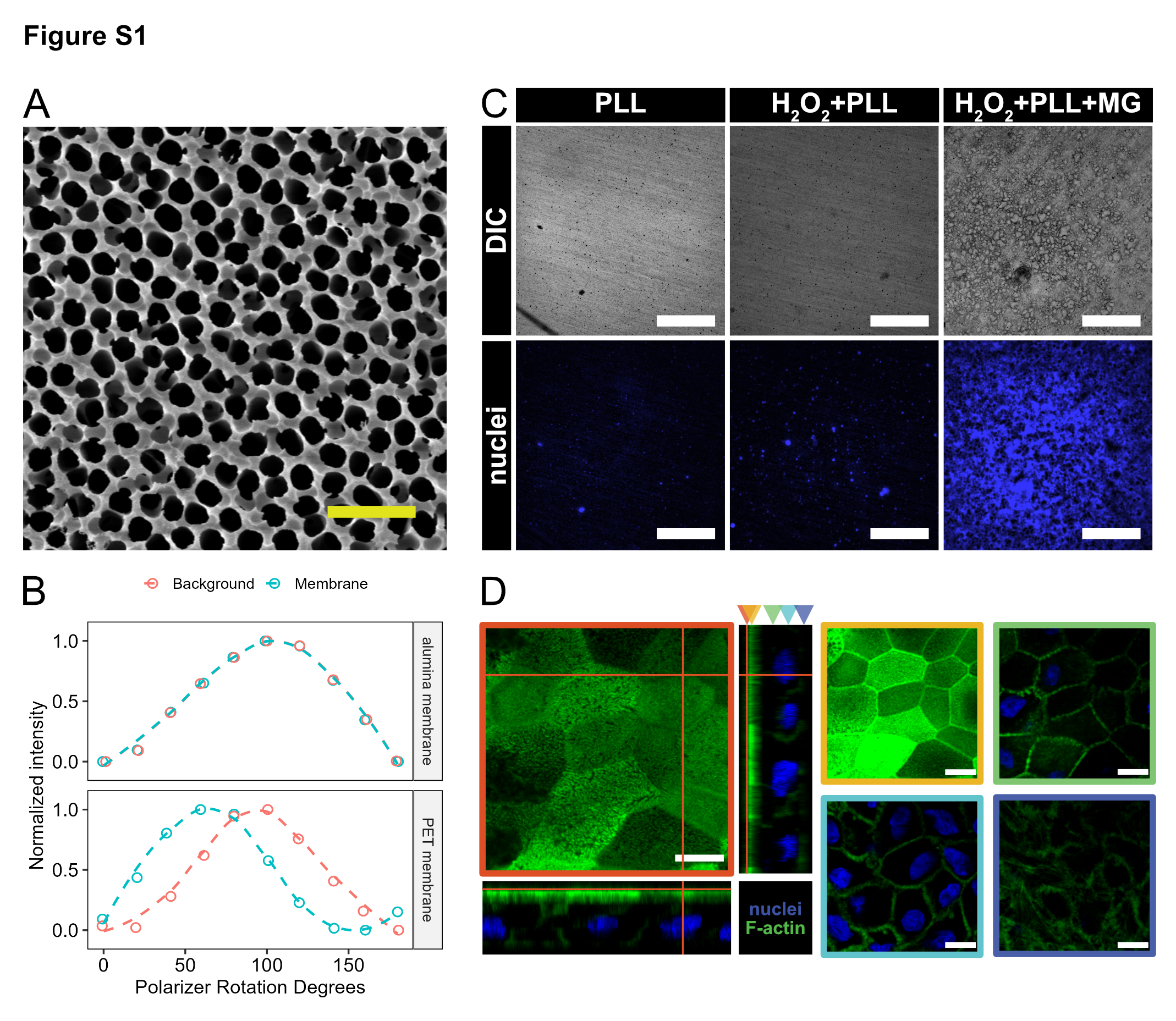

Supplement: FIG S1 [file mbio.00022-22-sf001.tif]

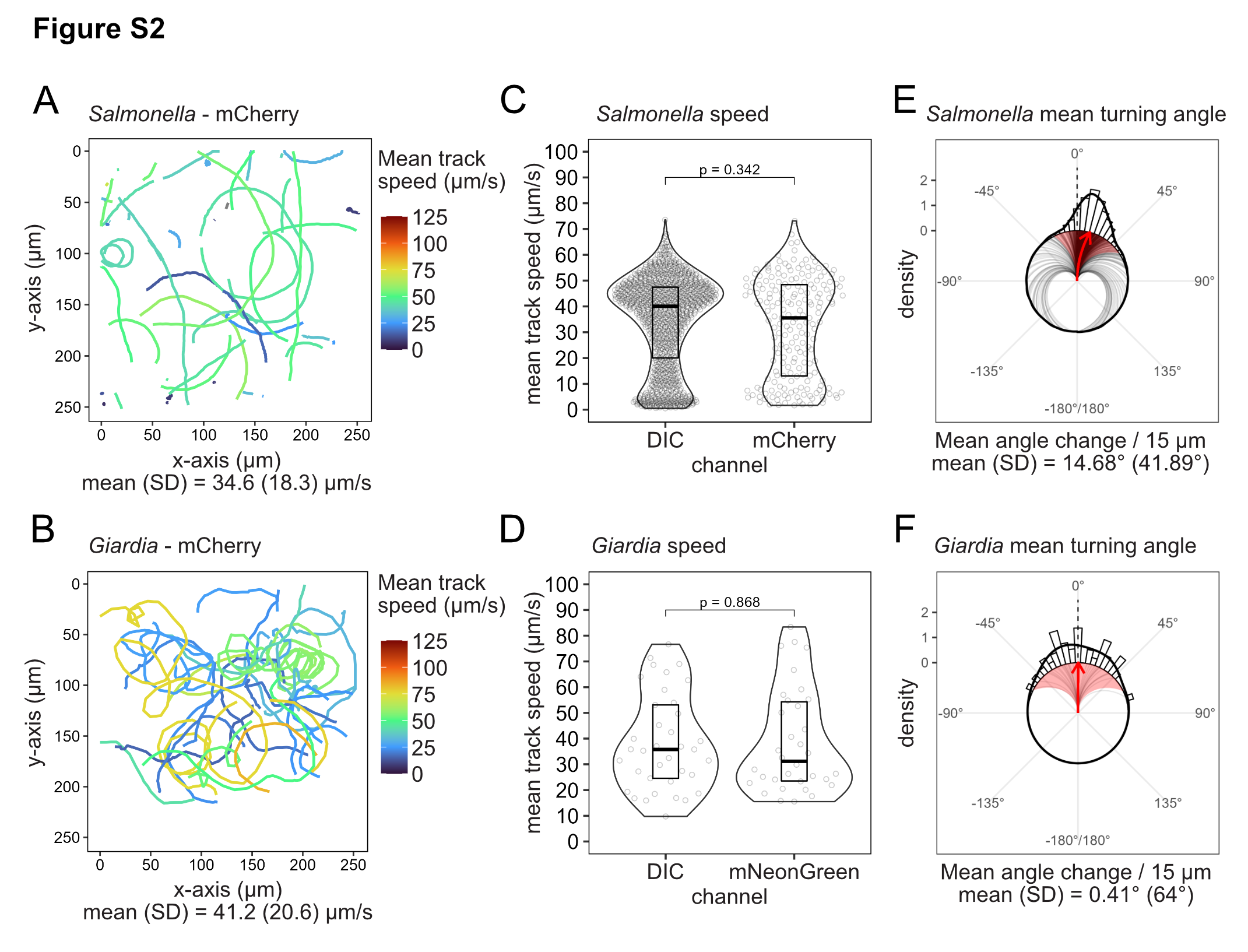

Supplement: FIG S2 [file mbio.00022-22-sf002.tif]

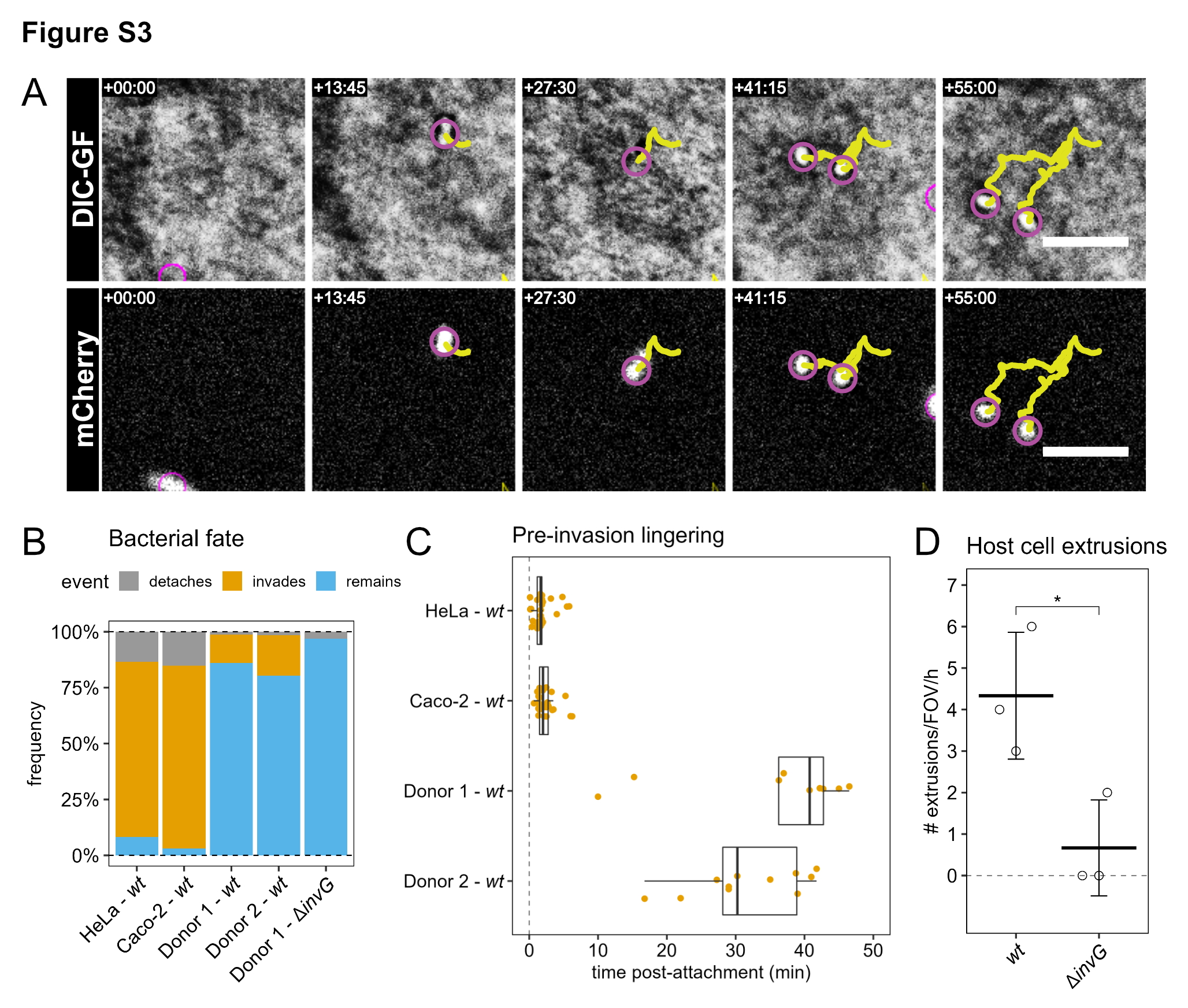

Supplement: FIG S3 [file mbio.00022-22-sf003.tif]

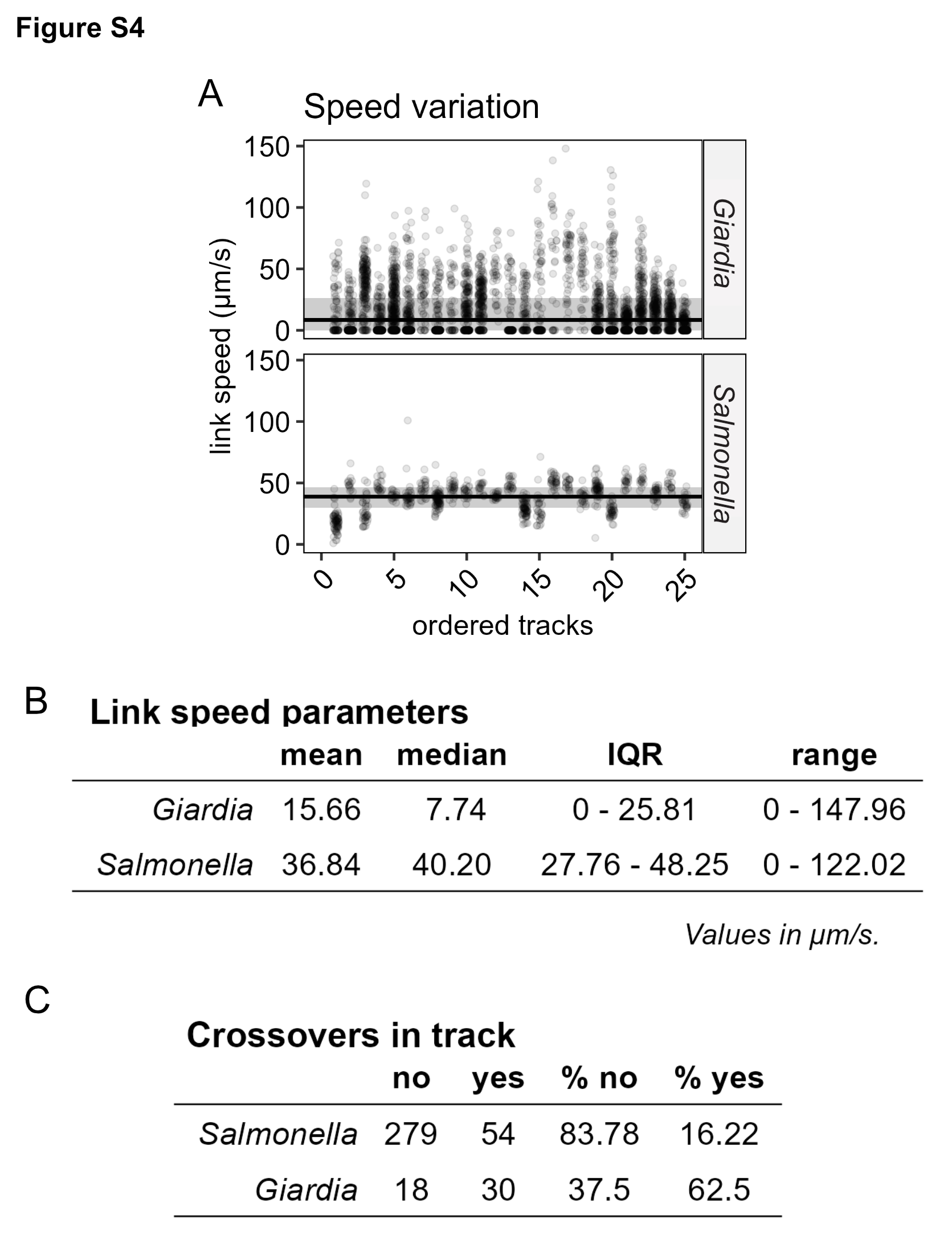

Supplement: FIG S4 [file mbio.00022-22-sf004.tif]
