# Supplementary material for: High-Definition DIC Imaging Uncovers Transient Stages of Pathogen Infection Cycles on the Surface of Human Adult Stem Cell-Derived Intestinal Epithelium
Source: mBio. 2022 Feb 1;13(1):e00022-22. doi: 10.1128/mbio.00022-22 (PMC8805028; doi:10.1128/mbio.00022-22)
Supplement: TABLE S1 [file mbio.00022-22-st001.pdf]

Table S1. Sequences of the primers used for the construction of the *Giardia-mNeonGreen* line.

Primer Name	Sequence (5' to 3')	Description
Fwd-p-BGiardin-XbaI	`ttctctagagtatgcagcac-tcacagagagatg`	Forward primer for Beta-Giardin promoter amplification including XbaI restriction site
Rv-p-BGiardin-mNeonGreen	`ctcctcgcccttgctcacca-tccttttattttcttaactt-gggctcaaat`	Reverse primer for Beta-Giardin promoter amplification including mNeonGreen overlap
Fwd-mNeonGreen	`atggtgagcaagggcgaggag`	Forward primer for mNeonGreen cds amplification
Rv-mNeonGreen	`tacttgtacagctcgtccat-gcc`	Reverse primer for mNeonGreen cds amplification
Fwd-3UTR-BGiardin-mNeonGreen	`ggcatggacgagctgtacaa-gtaagcgcctgcagtaaatc-atttac`	Forward primer for Beta-Giardin 3'UTR amplification including mNeonGreen overlap
Rv-3UTR-BGiardin-PacI	`cgtttaattaagtgctcaac-tgcaaccactac`	Reverse primer for Beta-Giardin 3'UTR amplification including PacI restriction site
